# Psychoactive Effects of *Lactobacillus johnsonii* Against Restraint Stress-Induced Memory Dysfunction in Mice Through Modulating Intestinal Inflammation and permeability—a Study Based on the Gut–Brain Axis Hypothesis

**DOI:** 10.3389/fphar.2021.662148

**Published:** 2021-05-26

**Authors:** Hesong Wang, Shunhui He, Jinge Xin, Tao Zhang, Ning Sun, Lianxin Li, Xueqin Ni, Dong Zeng, Hailin Ma, Yang Bai

**Affiliations:** ^1^ Guangdong Provincial Key Laboratory of Gastroenterology, Department of Gastroenterology, Institute of Gastroenterology of Guangdong Province, Nanfang Hospital, Southern Medical University, Guangzhou, China; ^2^ Department of Gastroenterology, Shunde Hospital, Southern Medical University, Foshan, China; ^3^ Animal Microecology Institute, College of Veterinary Medicine, Sichuan Agricultural University, Chengdu, China; ^4^ School of Science, Xihua University, Chengdu, China; ^5^ Plateau Brain Science Research Center, South China Normal University/Tibet University, Guangzhou, China

**Keywords:** *Lactobacillus johnsonii*, psychobiotics, gut-brain axis, memory dysfunction, intestinal environment

## Abstract

Though the underlying mechanism remains elusive, a close relationship between psychological stress and intestinal inflammation has been widely accepted. Such a link is very important to set the basis for our understanding of the critical role of gut-brain axis (GBA) in homeostatic processes in health and disease. Probiotics that could confer benefits to mental health through GBA are referred to as “psychobiotics”. This study aimed to further determine whether a potential psychobiotic strain, *Lactobacillus johnsonii* BS15 could prevent memory dysfunction in mice induced by psychological stress through modulating the gut environment, including intestinal inflammation and permeability. Memory dysfunction in mice was induced by restraint stress (RS), one of the most commonly utilized models to mimic psychological stress. The mice were randomly categorized into three groups including no stress (NS), restraint stress (RS), and probiotic (RS-P) and administered with either phosphate buffered saline (NS and RS groups) or *L. johnsonii* BS15 (RS-P group) every day from day 1–28. From days 22–28, the mice in RS and RS-P groups were subjected to RS each day. Results revealed that BS15-pretreatment enhanced the performance of RS-induced mice during three different behavioral tests for memory ability and positively modulated the hypothalamic–pituitary–adrenal axis by attenuating the serum corticosterone level. In the hippocampus, *L. johnsonii* BS15 positively modulated the memory-related functional proteins related to synaptic plasticity, increased neurotransmitter levels, and prevented RS-induced oxidative stress and mitochondria-mediated apoptosis. In the intestines, *L. johnsonii* BS15 protected the RS-induced mice from damaged gut barrier by enhancing the mRNA levels of tight junction proteins and exerted beneficial effects on the anti-inflammatory cytokine levels reduced by RS. These findings provided more evidence to reveal the psychoactive effect of *L. johnsonii* BS15 against memory dysfunction in RS-induced mice by modulating intestinal inflammation and permeability.

## Introduction

When provided in adequate amounts, probiotics could exert beneficial effects on the host (Sherman et al., 2009), such as improving the intestinal barrier function and gut microbiota, reducing proinflammatory cytokines, and increasing the intestinal antioxidant ability, and have been utilized for the prevention and/or treatment of many different intestinal diseases, such as inflammatory bowel disease (Wasilewski et al., 2015) and diarrhea (Selinger et al., 2013). However, the exact mechanism underlying these effects has not been identified (Guandalini et al., 2015; Sun et al., 2016). For example, Je et al., 2018 proved that ID-JPL934, a mixture of three probiotic strains (two *Lactobacillus* strains and one *Bifidobacterium* strain at a 1:1:1 ratio) attenuates dextran sulfate sodium-induced colitis by inhibiting the mRNA expression levels of proinflammatory cytokines in rodents. Probiotics also prevent or treat metabolic disorders and many other diseases (Le Barz et al., 2019). Given the close relationships between intestinal tracts and other organs, researchers have focused on expanding the application scope of probiotics.

Gut–brain axis (GBA) is defined as a network and communication among gastrointestinal tract, the enteric nervous system, and the brain. (Sudo et al., 2004) observed substantially high serum corticosterone level and reduced mRNA expression levels of brain-derived neurotrophic factor (BDNF) in the hippocampus and cortex in response to restraint stress (RS) in germ-free mice (born and fed entirely in the absence of microorganisms), indicating that commensal microbiota in the intestines could affect post-natal development. Effects on cognitive abilities including poor learning and memory and autism-like behavior were also found (Desbonnet et al., 2015; Vuong and Hsiao, 2017). The application of probiotic or prebiotic could lead to enhanced long-term potentiation (an experimentally evoked process in which the synaptic strength is rapidly increased and involves the crucial mechanism underlying learning and memory), increased BDNF concentrations, and improved intestinal immunity and barrier function, which consequently enhance the performance on a number of learning and memory tests (Zareie et al., 2006; Dash et al., 2015; Vazquez et al., 2015).

The association between intestinal environment and host behavior and the potential psychobiotics/probiotics that benefit mental health and yield positive psychiatric effects in psychopathology through GBA have been widely researched (Sarkar et al., 2018). Sgritta and colleagues (2019) reported consistent and robust reversal for social behavioral deficits by a potential psychobiotic, *Lactobacillus reuteri* in four different autistic spectrum disorder (ASD) mouse models (*Shank3B* ^−/–^ mice, valproic acid-treated mice, BTBR mice and germ-free mice). Lee et al., 2018 also found that *Lactobacillus plantarum* C29 could alleviate memory impairment in 5XFAD transgenic mice, indicating its possible ability to prevent Alzheimer’s disease. However, the limitation of psychobiotic researches should not be ignored that most reported findings about their effects opertains to rodent models rather than human studies. Also, most psychobiotic research findings are currently understood in terms of correlation rather than causation. Therefore, in order to make a potential psychobiotic strain convinceing enough to be applied in human studies, the mechanism underlying the beneficial effect must be considered to provide information for psychobiotic exploration based on GBA.

*Lactobacillus johnsonii* BS15 (CCTCC M2013663) was isolated from homemade yogurt from Hongyuan Prairie, Aba Autonomous Prefecture, China and was found to prevent non-alcoholic fatty liver disease by attenuating hepatic inflammation and mitochondrial injury and improving gut environment in obese mice (Xin et al., 2014). It also effectively prevents memory dysfunction induced by chronic high-fluorine intake by modulating the intestinal environment (Sun et al., 2020). Recently, we also found that *L. johnsonii* BS15 pretreatment enhanced intestinal health and prevented the hippocampus-related memory dysfunction induced by water avoidance stress (WAS), a well-established model for causing psychological stress (Wang et al., 2020). However, more evidence needs to be provided to prove whether or not *L. johnsonii* BS15 could be applied as a qualified psychobiotic that positively influences and protects mental health and cognitive behaviors against psychological stressors.

This study aimed to determine whether *L. johnsonii* BS15 could effectively prevent memory dysfunction in mice after restraint stress (RS) through modulating the gut environment, including intestinal inflammation and permeability. RS was induced in C57BL/6J mice to determine whether *L. johnsonii* BS15 could prevent memory dysfunction by conducting different behavioral tests. Given that the hippocampus is considered as a crucial brain region in memory ability and a neurobiological mediator underlying the bacteria-cognition link (Stachenfeld and Botvinick, 2017), the levels of memory-related functional proteins and neurotransmitters, antioxidant capacity, and apoptosis level were measured to reveal how *L. johnsonii* BS15 rescuing the impaired memory ability under RS influences the hippocampus. Intestinal integrity and inflammatory factors were also evaluated to further understand the mechanism on how *L. johnsonii* BS15 prevents hippocampus-related memory dysfunction.

## Materials and Methods

### Bacteria Preparation and Animal Treatment

*L. johnsonii* BS15 was maintained in de Man, Rogosa and Sharpe (MRS, QDRS Biotec, Qingdao, Shandong, China) broth under anaerobic environment at 37 C for 36 h. Heterotrophic plate count was used to evaluate the amount of bacterial cells. After collection, the bacterial cells were washed with saline and then suspended at pH 7.0 in phosphate buffered saline (PBS) at a concentration of 1 × 10^9^ cfu *L johnsonii* BS15/mL. Our previous experiment confirmed that oral gavage of *L. johnsonii* BS15 at the daily amount of 0.2 ml solution with 1 × 10^9^ cfu *L johnsonii* BS15/mL has the best preventive effects for obese mice (Xin et al., 2014).

A total of 108 5C7BL/6J male mice (3 week-old) were provided by Dashuo Biological Institute (Chengdu, Sichuan, China). The animals were fed on normal chow diet for 1 week before treatment to stabilize all metabolic conditions. All mice were housed with a 12 h light/dark cycle (lights on at 8:00 a.m. and off at 8:00 p.m.) in a room with strictly controlled temperature of 20–22 C and humidity of 40–60%. The animals were randomly divided into three groups each containing six cages (six mice per cage) and administered with either PBS (pH 7.0) (NS and RS groups) or *L. johnsonii* BS15 (RS-P group; daily amounts of 2 × 10^8^ cfu) through oral gavage from day 1–28. All animal experiments followed the guidelines for the use and care of laboratory animals (approval number: SYXKchuan 2019–187; approved by the Institutional Animal Care and Use Committee of Sichuan Agricultural University).

### Study Design and Sampling

The first day after 1 week stabilization was defined as day 1. From day 22–28, the mice in RS and RS-P groups were subjected to RS by placing them in 50 ml plastic conical centrifuge tubes for 60 min each day and directing their head toward the nasal end of the cylinder with air vents. The tubes restrained all physical movements without subjecting the animal to pain. All mice were not provided with food and water during the RS experiment.

After RS experiment in RS and RS-P groups on the morning of day 28, 8–10 mice from the three experimental groups were randomly selected and immediately sacrificed through cervical dislocation in accordance with institutional guidelines of animal care. Blood was collected through cardiac puncture, and the samples were immediately placed on ice and centrifuged. The isolated serum was frozen at −20 C until further analysis. The hippocampus and epithelial tissues of the jejunal and ileac samples were immediately removed from mice. After being washed by ice-cold sterilized saline, these samples were frozen in liquid nitrogen and then stored at −80 C. The other parts of hippocampus, the mucosa of jejunum and ileum were separately removed and frozen at −20 C until further analysis. The samples stored at −80 C were retrieved, and RNA of hippocampus, jejunum, and ileum was extracted using E. Z.N.A. Total RNA Kit (OMEGA Bio-Tek, Doraville, GA, United States) in accordance with the manufacturer’s guidelines. Total RNA (1 μg) was synthesized into first-strand complementary DNA (cDNA) using PrimeScriptTM RT reagent kit with gDNA Eraser (TaKaRa, Dalian, Liaoning, China). The cDNA products were stored at −20 C until subsequent tests. Another four mice in each group were sacrificed and their brain, jejunum and ileum was removed, fixed in 4% paraformaldehyde solution, and stored in 4 C for immunohistochemical and/or immunofluorescent assay.

**FIGURE 1:**
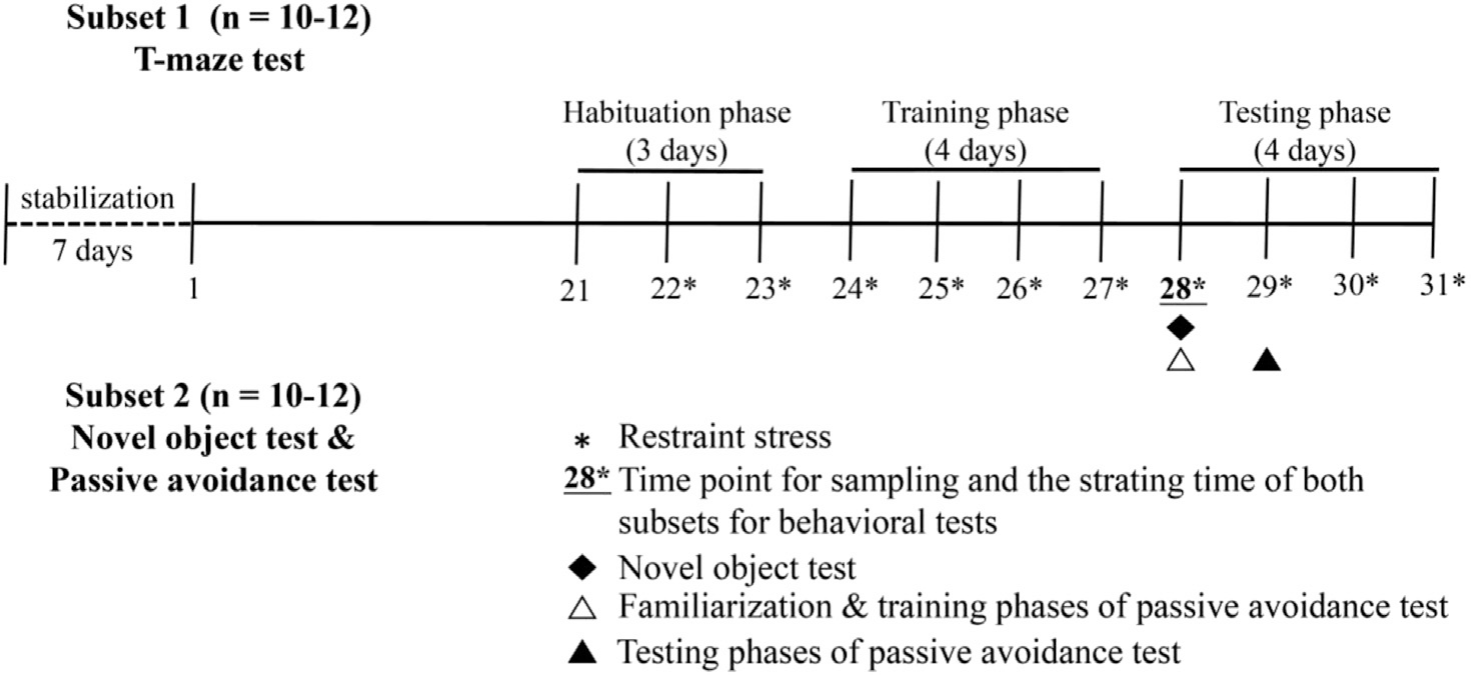
Flow diagram of behavioral tests.

In a subset of experiments, 10–12 mice from each group were selected for T-maze test. The habituation and training phases of T-maze test were performed at day 21 and lasted for 6 days until the 27th experimental day. On day 28, after the RS treatment for RS and RS-P groups, the testing phase of T-maze test was conducted for all three experimental groups. Another 10–12 mice from each group were selected for novel object and passive avoidance tests. Novel object test was carried out on day 28 after the mice were subjected to RS, except mice in NS group. The mice were allowed to have a 10 min rest period between two tests and then placed in the wooden box for passive avoidance test to experience familiarization and training. The testing phase for passive avoidance test was conducted on day 29. All the mice that underwent behavioral tests were not selected to avoid the carry-over effects of behavioral testing on inflammation related parameters and other biomarkers (Boitard et al., 2014; Beilharz et al., 2017). Figure 1 displays the flow diagram of behavioral tests applied in this study.

### Behavioral Tests

#### Novel Object Test

Mice have the tendency to investigate a novel object rather than a familiar one. On this basis, novel object test was undertaken for hippocampus-dependent memory formation following the method described by Gareau et al., 2011 with minor modification. The test was briefly described below:

The mice without RS were placed into a dark open-field arena (40 × 40 × 45 cm, l×b×h) and allowed to freely explore for 1 h for habituation. The following two different objects were exposed to the mice after RS or habituation: blue and orange tube caps with the same size and shape and a smooth pebble of proper size. Behavioral assessment consisted of the following two phases:

### Familiarization Phase

The mice were placed into the arena with a blue tube cap and an orange tube cap placed in opposite corners and allowed to freely explore the two objects for 5 min. The objects were then removed, and the mice were given a rest period (20 min) before the testing phase.

### Testing Phase

The orange tube cap was replaced by the smooth pebble during the rest period, and the mice after resting were re-exposed to the blue tube cap and the smooth pebble. Memory could be evaluated as the frequency to explore the smooth pebble. compared with the blue tube cap during the testing phase. Exploration ratio represents the smelling bouts proportion related to the new object vs. the old one (ratio of the frequency of smelling the smooth pebble to the total frequency of smelling the blue tube cap and the smooth pebble). A ratio of 0.5 represents impaired hippocampus-dependent memory as no discrimination is found between the two objects. In the present test, exploration was defined by orientating the mice toward the object with the nose pointing directly to the object within 1–2 cm.

### Passive Avoidance Test

Memory acquisition and retention were evaluated using step-down passive avoidance test. The apparatus was a wooden box (40 × 40 × 40 cm, l×b×h) with floor consisted of 0.3 cm caliber stainless paralleled steel bars. The bars were spaced 1 cm apart. A small platform (4 × 4 × 4 cm, l×b×h) was placed in the center of the grid floor.

### Familiarization and Training Phases

For familiarization, the mice placed on the platform in the wooden box were allowed to freely explore for 3 min. The training phase was then started after the mice became familiar with the apparatus. In the training phase, the mice were gently placed on the platform (3 min) and immediately received 2 s of electric shock (36 V, 1 mA, 50 Hz) once they stepped down with all four paws on the grid. Afterward, the animals generally returned to the platform.

### Testing Phase

The testing phase (3 min) was started 24 h after the training phase with identical process, and RS was induced in advance when needed. Escape latency was recorded as the duration the mice first stayed on the platform. Error number was measured as the repeated times the mice stepped down on the grid during the phase (3 min) (Malekmohamadi et al., 2007). Poor memory ability could be indicated by short escape latency and high error numbers (Chen et al., 2014).

### T-Maze Test

The applied T-maze was a “T”-shape enclosed apparatus with a start arm (60 × 10 × 20 cm, l×b×*h*) and two goal arms (30 × 10 × 20 cm, l×b×h). Given their natural tendency to explore a novel environment, the mice were first placed at the base of the start arm and allowed to freely enter one of the goal arms. A mouse tends to choose the other goal arm which is not visited prior to the second trial, and this phenomenon could reflect the memory of the first choice. Alternation is sensitive to memory dysfunction (especially hippocampus-related) and represents a model of working memory (Albert-Gasco et al., 2017).

Rewarded T-maze test was chosen (over spontaneous T-maze test) because mice could run many trials per day before getting sated (Deacon and Rawlins, 2006). As food reward, 0.07 ml of 1:1 (vol/vol) full fat/water sweetened condensed milk (Nestle, Qingdao, Shandong, China) mixture was given per trial by preset pipette. The test was briefly described below:

### Habituation and Training Phases

During habituation phase (3 days), the mice were softly stroked, slowly picked up, and put down three times per day (3 min each time) to ensure that they were accustomed to the touch from operators. Given that they are wary of eating anything new (Forestier et al., 2018), the mice were fed 0.5 ml of food reward each day to get familiar with its taste. Training phase (4 days) was performed after habituation, and the animals were placed into T-maze with all arms open and allowed to explore freely for 10 min. The mice in the start arm were allowed to run toward one goal arm, and their reward was provided into the food well while the other arm was blocked by its door. No more than 3 min of training time was given until the mice discovered that the well was empty. Each mouse was trained four times per day (left and right runs were given with equal numbers).

### Testing Phase

Each mouse was tested for 4 days with five trials per day. The rodent was allowed to explore the whole maze without loss of interest. RS was given before the test started each day when needed. With one of the goal arms blocked (randomly chosen for each trial), the mice from the start arm were allowed to run toward the open goal arm with consumable reward. They were immediately returned to the start arm as soon as they found the well empty, and the operator opened the door of the blocked goal arm. With 0 s (for trails at day 1 and 2) or 1 min (for trails at day 3 and 4) of retention interval, the mice were allowed to run from the start arm again and choose one arm. If it chose the correct arm, then the mouse was allowed to consume the reward; if incorrect, the mouse was removed after definitively discovering that the well was empty. Working memory with 0 s and 1 min of retention interval was separately assessed as a ratio of correct times to total trail times (*n* = 10).

### Biochemical Analysis

Corticosterone and D-lactate serum contents and diamine oxidase (DAO) activity were quantified using ELISA) kits specific for mice (MLBIO Biotechnology Co., Ltd, Shanghai, China) following the manufacturer’s instructions. The standard curve was used to calculate the contents of determined proteins. In the hippocampus, the contents of neurotransmitters (dopamine, DA; 5-hydroxytryptamine, 5-HT; acetylcholine, Ach; glutamic acid, Glu; gamma-aminobutyric acid, GABA; nitric oxide, NO), neurotransmitter-related proteins (including nitric oxide synthase, NOS; acetyl-cholinesterase, AchE; choline acetyltransferase, ChAT), and two apoptosis-related proteins (Bax and Bcl-2) were determined by ELISA using reagent kits specific for mice (MLBIO Biotechnology Co., Ltd, Shanghai, China) in accordance with the manufacturer’s instructions. The antioxidant indexes in the hippocampus were measured using commercial kits (Jiancheng Bioengineering Institute, Nanjing, Jiangsu, China), including the activities of catalase (CAT), total antioxidation capacity (T-AOC), glutathione peroxidase (GSH-Px), superoxide dismutase, and malondialdehyde (MDA) and GSH contents (Zareie et al., 2006). Inflammatory factors contents in the jejunum and ileum were also determined by ELISA using reagent kits specific for mice (MLBIO Biotechnology Co., Ltd, Shanghai, China). The determined inflammatory factors included interleukin (IL)-1β, IL-4, IL-6, IL-10, tumor necrosis factor-alpha (TNF-α), and interferon-gamma (IFN-γ).

### Immunohistochemistry

Tissues for immunohistochemical assay were embedded by paraffin and cut by a microtome. Using a microwave oven (model: P70D20TL-P4; Galanz, Guangdong, China), slices were submerged in citrate antigen retrieval solution and heated at medium heat until boiling. The temperature was then ceased and tissues were kept warm for 8 min. The tissues were heated at medium–low heat for 7 min. The slices after free cooling were placed into PBS (pH 7.4) and shaken for 5 min for decoloration, which was repeated three times. The sections were then incubated in 3% oxydol for 25 min at room temperature and away from the light to block endogenous peroxidase. The slices were washed three times in PBS by shaking for 5 min, then sealed for 30 min by 3% bull serum albumin, and incubated with monoclonal rabbit anti-BDNF (1:400) or polyclonal rabbit anti-CREB (1:500) antibodies at 4°C overnight. Species-specific biotinylated anti-rabbit immunoglobulin (horseradish peroxidase labeled) was used for immuno-detection. Following the second antibody incubation, the 3,3′-diaminobenzidine staining kit was used to complete the reaction according to the manufacturer’s instructions. Hematoxylin staining was performed to re-stain the nucleus.

### Immunofluorescence

The 5-µm-thick paraffin-embedded jejunal and ileal tissue sections were used for immunofluorescence. Heat-induced antigen retrieval was performed by autoclaving the sections for 10 minutes at 121°C in 10mM sodium citrate buffer (pH 6.0). The sections were blocked with 8% skim milk in TBST at 37°C for 40 minutes, and then immunostained using primary antibodies against ZO-1 (1:200, GB11195, rabbit; Servicebio), occludin (1:200, GB111401, rabbit; Servicebio), claudin-1 (1:200, GB11032, rabbit; Servicebio) at 4°C overnight. The sections were washed and incubated with secondary fluorescent antibodies at 37°C for 60 minutes. The secondary antibodies was CY3 goat anti-rabbit IgG (1:300; GB21303; Servicebio). Sections were mounted with Nikon DS-U3 with DAPI (G1012, Servicebio). Images were captured with an Nikon Eclipse C1 fluorescence microscope (Nikon, Tokyo, Japan).

### Real-Time Quantitative Polymerase Chain Reaction (qPCR) Analysis

PCR was performed to determine the prepared cDNA products from the hippocampus, jejunum, and ileum. A CFX96 Real-time PCR Detection System (Bio-Rad, Hercules, CA, United States) with iTaq Universal SYBR Green Supermix (Bio-Rad, Hercules, CA, United States) was used with the following protocol: 5 min at 95 C, 40 cycles of 10 s denaturation at 95 C, and 30 s annealing/extension at optimum temperature (Tables 1, 2). PCR product purity was monitored by a final melting curve analysis. Standard curves were obtained through serial dilution. The primer sequences for targeted genes are presented in Table 1. _ΔΔ_Ct method was applied to estimate mRNA abundance. The samples (*n* = 6) in each group were analyzed in triplicate, and Ct was calculated as (Ct_target_ − Ct_β-actin_)_treatment_ − (Ct_target_ − Ct_β-actin_)_control._ β-actin was used as the eukaryotic housekeeping gene to normalize relative gene expression levels. Mean values of measurements were applied to evaluate the mRNA expression levels of cyclic amp (cAMP) response element binding protein (CREB), N-methyl-D-aspartate receptor (NMDAR), brain-derived neurotrophic factor (BDNF), c-Fos, stem cell factor (SCF), neural cell adhesion molecule (NCAM), Bcl-2, Bad, Bcl-xL, Bax, caspase-3 and caspase-9 in the hippocampus and IL-1β, tumor necrosis factor (TNF)-α, interferon (IFN)-γ, IL-4, IL-10, IL-6, claudin-1, occludin, and zoluna occludens protein (ZO)-1 in the jejunum and ileum.

**TABLE 1:** Primer sequences for RT-qPCR in hippocampus.

Gene	Tm (°C)	Sequence	References
β-actin	60	F: GCT​CTT​TTC​CAG​CCT​TCC​TT	Sun et al. (2020)
		R: GATGTCAACGTCACACTT	
BDNF	60	F:GCGCCCATGAAAGAAGTAAA	Niu et al. (2018)
		R: TCG​TCA​GAC​CTC​TCG​AAC​CT	
c-Fos^a^	59.5	F:CAGAGCGGGAATGGTGAAGA	—
		R:CTGTCTCCGCTTGGAGTGTA	
NCAM	60	F: GGG​AAC​TCC​ATC​AAG​GTG​AA	Niu et al. (2018)
		R: TTG​AGC​ATG​ACG​TGG​ACA​CT	
SCF	60	F:CCTTATGAAGAAGACACAAACTTGG	Niu et al. (2018)
		R:CCATCCCGGCGACATAGTTGAGGG	
CREB	60	F: CCA​GTT​GCA​AAC​ATC​AGT​GG	Niu et al. (2018)
		R: TTG​TGG​GCA​TGA​AGC​AGT​AG	
NMDAR	60	F: GTGGATTGGGAGGATAGG	Niu et al. (2018)
		R: TTAGTCGGGCTTTGAGG	
Caspase-9	61	F: GAG​GTG​AAG​AAC​GAC​CTG​AC	Guo et al. (2017)
		R: AGA​GGA​TGA​CCA​CCA​CAA​AG	
Caspase-3	59	F: ACA​TGG​GAG​CAA​GTC​AGT​GG	Guo et al. (2017)
		R: CGT​CCA​CAT​CCG​TAC​CAG​AG	
Bax	61	F: ATGCGTCCACCAAGAAGC	Guo et al. (2017)
		R: CAG​TTG​AAG​TTG​CCA​TCA​GC	
Bad	60	F: AGA​GTA​TGT​TCC​AGA​TCC​CAG	Guo et al. (2017)
		R: GTC​CTC​GAA​AAG​GGC​TAA​GC	
Bcl-2	61	F: AGC​CTG​AGA​GCA​ACC​CAA​T	Guo et al. (2017)
		R: AGC​GAC​GAG​AGA​AGT​CAT​CC	
Bcl-xl	62	F: TGT​GGA​TCT​CTA​CGG​GAA​CA	Guo et al. (2017)
		R: AAG​AGT​GAG​CCC​AGC​AGA​AC	

a The primer sequences of c-fos is designed by National Center for Biotechnology Information (NCBI) and the referenced gene ID is 14,281.

**TABLE 2:** Primer sequences for RT-qPCR in small intestines.

Gene	Tm (°C)	Sequence	References
β-actin	60	F: GCT​CTT​TTC​CAG​CCT​TCC​TT	Sun et al. (2020)
		R: GATGTCAACGTCACACTT	
Claudin-1	60	F:GGGGACAACATCGTGACCG	Liu et al. (2017)
		R:AGGAGTCGAAGACTTTGCACT	
Occludin	60	F:TTGAAAGTCCACCTCCTTACAGA	Liu et al. (2017)
		R:CCGGATAAAAAGAGTACGCTGG	
ZO-1	60	F:GATCCCTGTAAGTCACCCAGA	Liu et al. (2017)
		R:CTCCCTGCTTGCACTCCTATC	
TNF-α	59.0	F:ACGGCATGGATCTCAAAGAC	Xin et al. (2014)
		R:AGATAGCAAATCGGCTGACG	
IL-1β	60	F:ATGAAAGACGGCACACCCAC	Liu et al. (2017)
		R:GCTTGTGCTCTGCTTGTGAG	
IL-6	60	F:TGCAAGAGACTTCCATCCAGT	Liu et al. (2017)
		R:GTGAAGTAGGGAAGGCCG	
IFN-γ	53	F:TCAAGTGGCATAGATGTGGAAGAA	Liu et al. (2017)
		R:TGGCTCTGCAGGATTTTCATG	
IL-10	56	F:GGTTGCCAAGCCTTATCGGA	Liu et al. (2017)
		R:ACCTGCTCCACTGCCTTGCT	
IL-4	55	F:ACAGGAGAAGGGACGCCAT	Usuda et al. (2012)
		R:GAAGCCCTACAGACGAGCTCA	

### qPCR Quantification

The population of total bacteria and *Lactobacillus johnsonii* was estimated in the jejunum and ileum following the method of Xin and colleagues (2014). A CFX Connect™ real-time system (Bio-Rad, Hercules, CA, United States) and SYBR® Premix Ex Taq™ II (TaKaRa, Dalian, Liaoning, China) were used to perform qPCR. Table 3 presents the primers for the qPCR of the microbiota. The reaction mixture (25 μL) included SYBR® Premix Ex TaqTM II (12.5 μL), forward and reverse primers (1 μL), sterile deionized water (9.5 μL), and DNA template (1 μL). PCR was performed as follows: 95 C for 1 min, 40 cycles of 94 C for 15 min, and annealing at optimal temperatures for 30 s at 72 C. The specificity of the PCR primers was regulated by generating melting curves.

**TABLE 3:** Primer information on the microflora for qPCR.

Target species	Tm (°C)	Primer sequence (5→3)	References
Total bacteria	60.0	F: CGGYCCAGACTCCTACGGG	Xin et al. (2014)
		R: TTA​CCG​CGG​CTG​CTG​GCA​C	
*L. johnsonii*	61.4	F:CACTAGACGCATGTCTAGAG	Xin et al. (2014)
		R:AGTCTCTCAACTCGGCTATG	

### Data Analysis

Data were analyzed based on individual mice. Statistical analysis was performed using one-way ANOVA, followed by Duncan’s multiple-range test for multiple comparisons (both normality test and equal variance test passed) (SigmaPlot for Social Sciences version 12). Differences at *P* < 0.05 were considered statistically significant.

## Results

### Behavioral Tests

**FIGURE 2:**
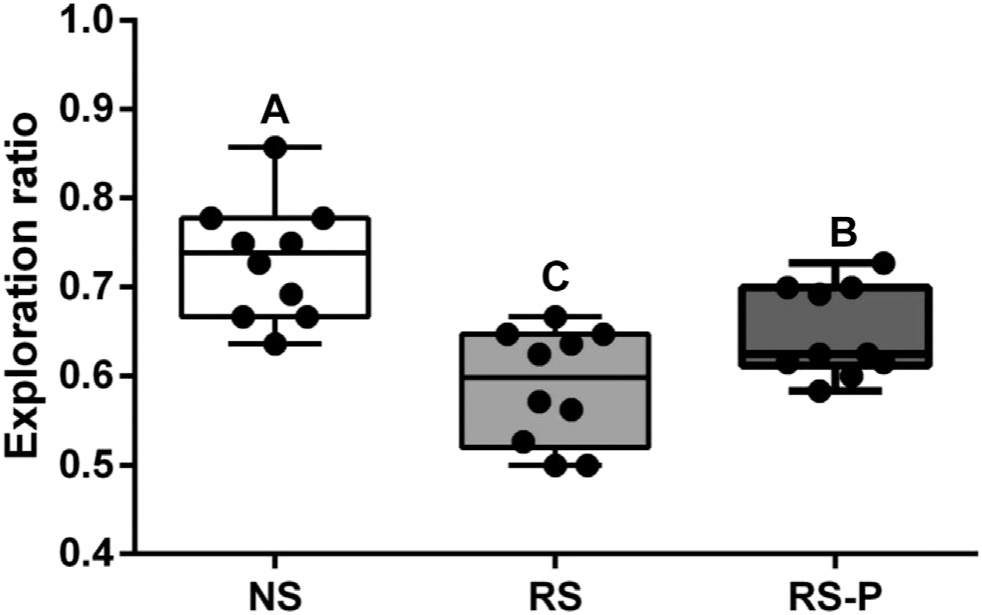
Effects of *L. johnsonii* BS15 on exploration ratio by novel object test. Data are presented with the means ± standard deviation (*n* = 10). Bars with different letters are significantly different on the basis of Duncan’s multiple range test (*P* < 0.05).

**FIGURE 3:**
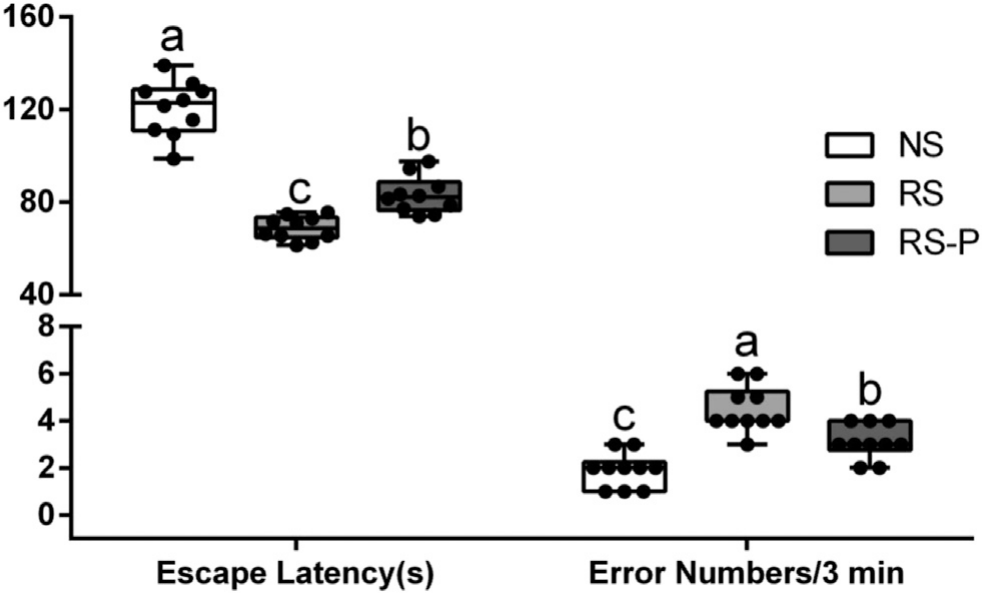
Effects of *L. johnsonii* BS15 on the escape latency and error numbers by passive avoidance test. Data are presented with the means ± standard deviation (*n* = 10). Bars with different letters are significantly different on the basis of Duncan’s multiple range test (*P* < 0.05).

**FIGURE 4:**
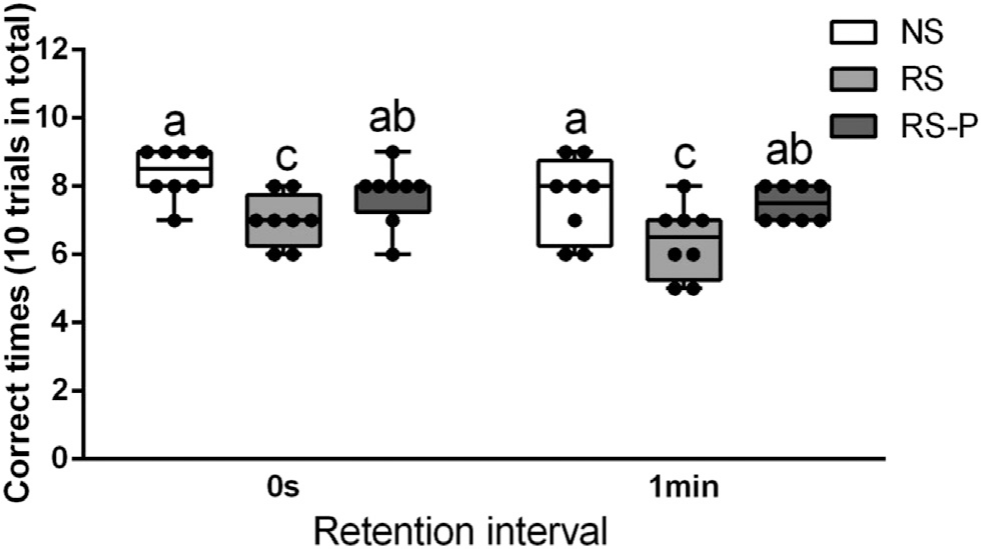
Effects of *L. johnsonii* BS15 on the correct times with both 0s and 1min of retention interval by T-maze test. Data are presented with the means ± standard deviation (*n* = 8). Bars with different letters are significantly different on the basis of Duncan’s multiple range test (*P* < 0.05).

Figures 2–4 show the results of behavioral tests for memory abilities. Significantly lower time in exploration ratio (Figure 2) and escape latency (Figure 3) were observed (*P* < 0.05) in the RS group compared with those in the NS group. Correct times for 0 s and 1 min of retention interval (Figure 4) in the RS group were also significantly lower (*P* < 0.05) than those in the NS group. Meanwhile, error numbers (Figure 2) in the RS group were significantly higher than those in the NS group. Positive changes in all indexes were induced by *L. johnsonii* BS15 in the RS and RS-P groups. In particular, the exploration ratio (Figure 2), escape latency (Figure 3), and correct times for both 0 s and 1 min of retention interval (Figure 4) were significantly high (*P* < 0.05) in the RS-P group. The error numbers (Figure 3) in the RS group were significantly lower (*P* < 0.05) than those in the RS-P group. Moreover, significant differences (*P* < 0.05) in exploration ratio (Figure 2), escape latency, and error numbers (Figure 3) were observed between the RS-P and NS groups, whereas the correct times showed no significance (*P* > 0.05) (Figure 3).

### Serum Corticosterone and Memory-Related Functional Proteins

**FIGURE 5:**
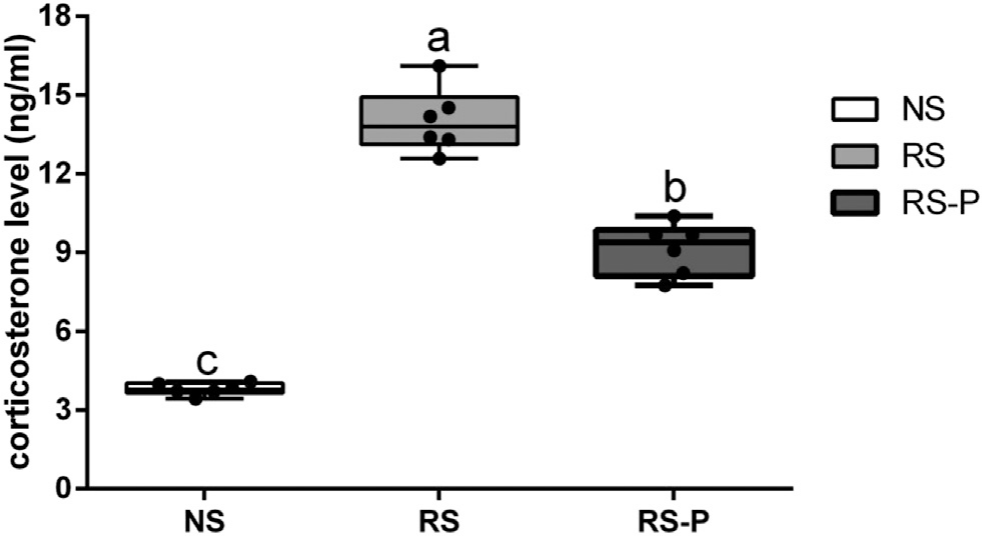
Levels of corticosterone in the serum. Data are presented with the means ± standard deviation (*n* = 6). Bars with different letters are significantly different on the basis of Duncan’s multiple range test (*P* < 0.05).

**FIGURE 6:**
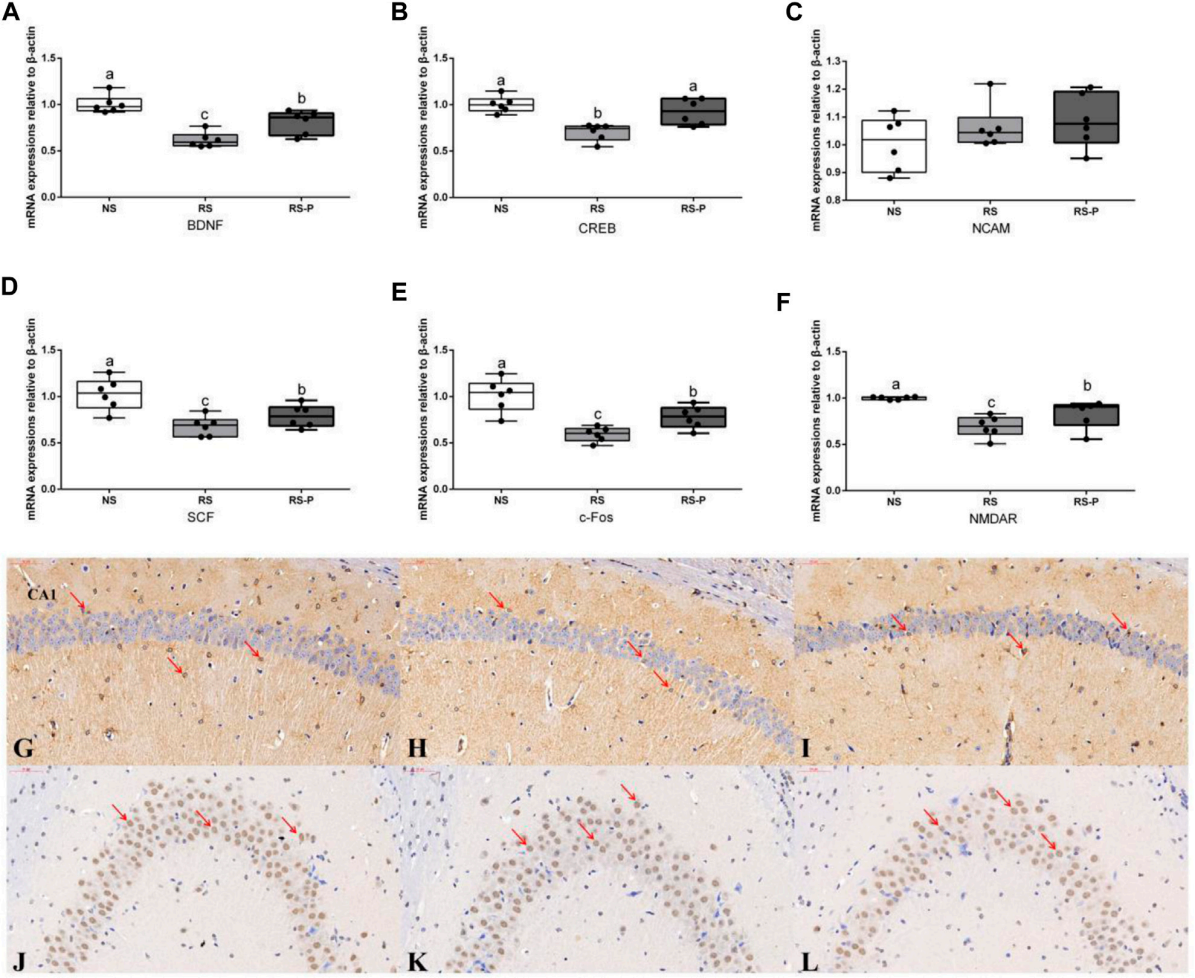
Expression levels of memory-related functional proteins in the hippocampus. **(A)–(F)**: Relative expression of BDNF, CREB, NCAM, SCF, c-Fos, and NMDAR, respectively. Data are presented with the means ± standard deviation (*n* = 6). Bars with different letters are significantly different on the basis of Duncan’s multiple range test (*P* < 0.05). **(G)–(I)** Immunohistochemistry of BDNF expressions in the hippocampus of NS **(G)**, RS **(H)** and RS-P **(I)** groups. **(J)–(L)** Immunohistochemistry of CREB expressions in the hippocampus of NS **(J)**, RS **(K)** and RS-P **(L)** groups. The BDNF- and CREB-positive cells are brown like the arrow indication, the magnification of all the figures are 40×. BDNF, brain-derived neurotrophic factor; CREB, cyclic ampresponse element binding protein; NCAM, neural cell adhesion molecule; SCF, stem cell factor; NMDAR, N -methyl-D-aspartate receptor.

Figure 5 shows the differences in corticosterone levels in the serum among three groups. Corticosterone level was significantly higher (*P* < 0.05) in the RS group compared with that in the other groups, and that in the RS-P group was significantly higher (*P* < 0.05) than that in the NS group. Levels of memory-related functional proteins are shown in Figure 6. All indexes were significantly lower (*P* < 0.05) in the RS group than in the NS group (Figures 6A–F). Although no significant change (*P* > 0.05) in NCAM (Figure 6C) was observed between the RS and RS-P groups, the mRNA expression levels of BDNF, CREB, SCF, c-Fos, and NMDAR in RS-P group were significantly higher (*P* < 0.05) than those in the RS group (Figures 6A,B,D–F). Except for CREB with no significant difference (*P* > 0.05), all other indexes in the RS-P group were significantly lower (*P* < 0.05) than those in the NS group. As shown in Figure 6G–L, the protein expression levels of BDNF and CREB were significantly reduced in the RS group compared with that in the other two groups.

### Neurotransmitters and Related Functional Proteins

**FIGURE 7:**
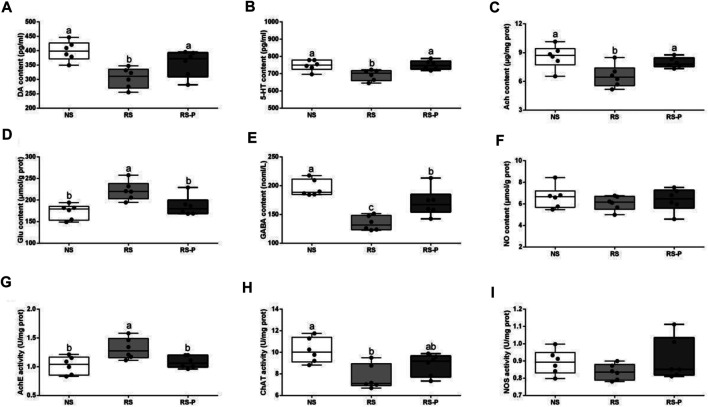
Levels of neurotransmitters in the hippocampus. Data are presented with the means ± standard deviation (*n* = 6). Bars with different letters are significantly different on the basis of Duncan’s multiple range test (*P* < 0.05). **(A)–(F)**: Levels of DA, 5-HT, Ach, Glu, GABA and NO, respectively; **(G)—(I)**: Activities of AchE, ChAT and NOS, respectively. DA, dopamine; 5-HT, 5-hydroxytryptamine; Ach, acetylcholine; Glu, glutamic acid; GABA, gamma-aminobutyric acid; NO, nitric oxide; AchE, acetyl-cholinesterase; ChAT, choline acetyltransferas; NOS, nitric oxide synthase.

Figures 7A–C show that although DA, 5-HT, and Ach levels were significantly lower in the RS group than those in the NS group, they were significantly increased (*P* < 0.05) by *L. johnsonii* BS15 in RS-P group and showed no significant differences (*P* > 0.05) compared with those in the NS group. In addition, the Glu content (Figure 7D) and AchE activity (Figure 7G) in the RS group were significantly higher (*P* < 0.05) than those in the other groups but were influenced by *L. johnsonii* BS15 to show no significant difference (*P* > 0.05) between the NS and RS-P groups. As shown in Figure 7E, the GABA content in the RS-P group was significantly higher (*P* < 0.05) than that in the RS group but lower (*P* < 0.05) than that in the NS group. As shown in Figure 7H, the ChAT activity in the RS group was significantly decreased (*P* < 0.05) compared with that in the NS group but was not significantly different (*P* > 0.05) in the RS-P group compared with the NS or RS group. The levels of NO and NOS activity (Figures 7F,I) were not significantly influenced (*P* > 0.05) by BS15 and RS.

### Antioxidant Capacity and Apoptosis

**FIGURE 8:**
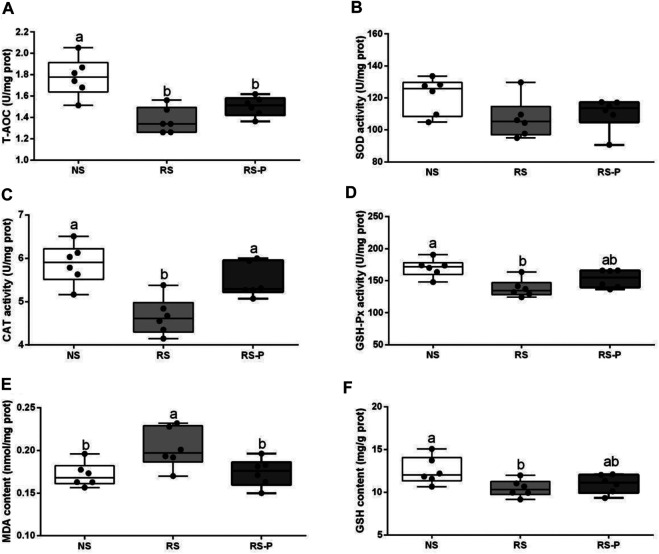
Antioxidant indexes in the hippocampus. Data are presented with the means ± standard deviation (*n* = 6). Bars with different letters are significantly different on the basis of Duncan’s multiple range test (*P* < 0.05). **(A)–(F)**: Activities or contents of T-AOC, SOD, CAT, GSH-Px, MDA and GSH, respectively. T-AOC, total antioxidation capacity; SOD, superoxide dismutase; CAT, catalase; GSH-Px, glutathione peroxidase; MDA, malondialdehyde; GSH, glutathion.

**FIGURE 9:**
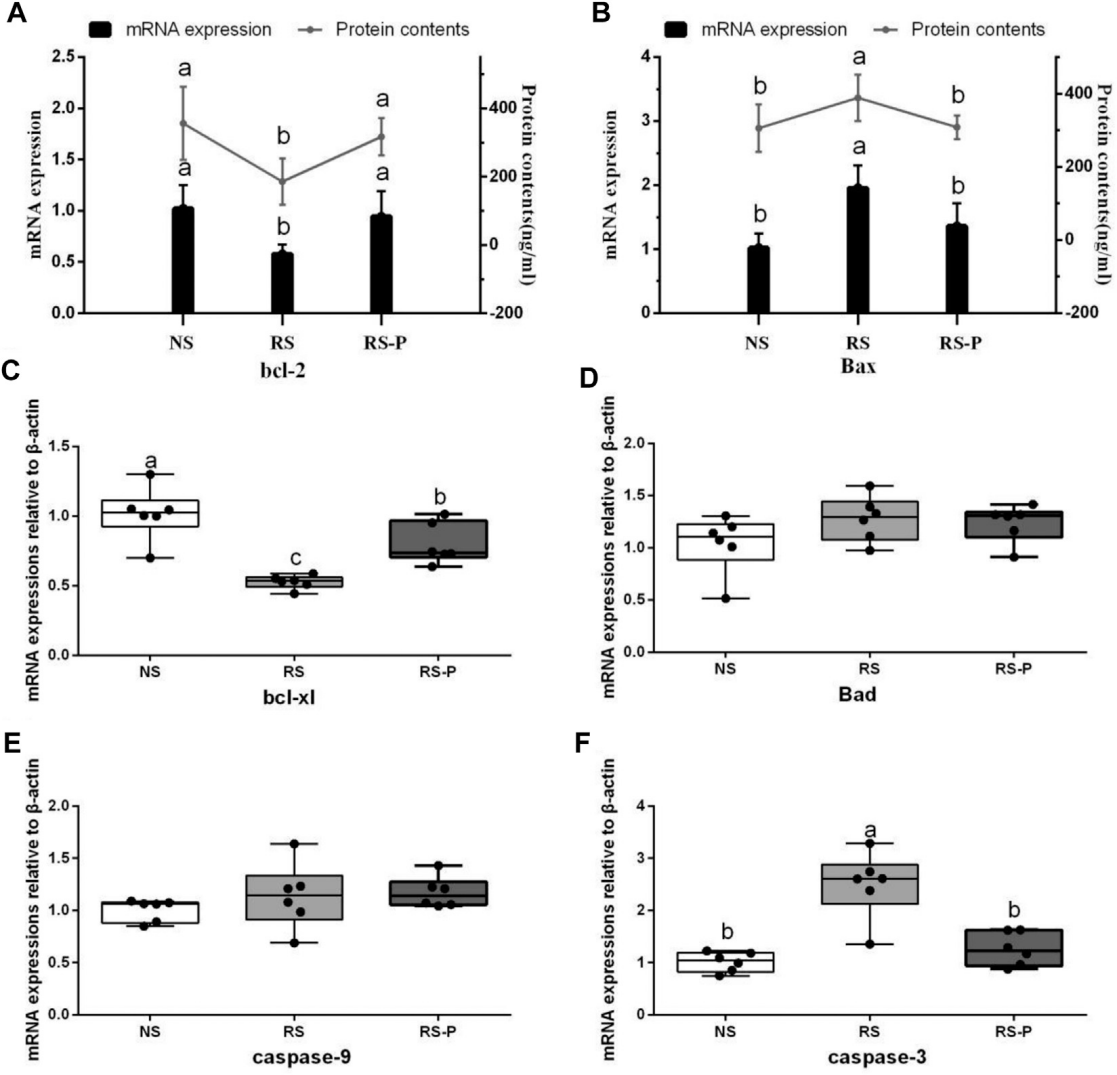
Apoptosis-related functional protein contents and mRNA expression levels in the hippocampus. Data are presented with the means ± standard deviation (*n* = 6). Bars with different letters are significantly different on the basis of Duncan’s multiple range test (*P* < 0.05). **(A)–(B)**: mRNA expression levels and protein contents of bcl-2 and Bax; **(C)—(F)**: mRNA expression levels of bcl-xl, Bad, caspase-9 and caspase-3, respectively.

Figure 8 demonstrates antioxidant indexes in the hippocampus. As shown in Figure 8A, T-AOC was significantly lower (*P* < 0.05) in the RS and RS-P groups than that in the NS group, but no difference was shown (*P* > 0.05) between the two RS groups. No changes were observed (*P* > 0.05) in SOD activity (Figure 8B). Figures 8C,E show significantly lower CAT activity (*P* < 0.05) and higher MDA content (*P* < 0.05) in the RS group than in the other two groups, but no significant differences of these two indexes were found (*P* > 0.05) between the NS and RS-P groups. Meanwhile, GSH-Px activity and GSH content were significantly low (*P* < 0.05) in the RS-P group (Figures 8D,F) but showed no differences (P > 0.05) compared with those in the NS or RS group. The results of apoptosis-related functional protein contents and mRNA expression levels in the hippocampus are presented in Figure 9. Significantly lower values of bcl-2 protein content (Figure 9A) and mRNA expression levels of bcl-2 (Figure 9A) and Bcl-xL (Figure 9C) were found (*P* < 0.05) in the RS groups than those in the NS and RS-P groups. Higher protein and mRNA expression levels of Bax (Figure 9B) and caspase-3 (Figure 9F) were also found (*P* < 0.05) in the RS groups. These indexes showed no significant differences (*P* > 0.05) between the NS and RS-P groups. However, the mRNA expression levels of Bad (Figure 9D) and caspase-9 (Figure 9E) remained unchanged (*P* > 0.05).

### Intestinal Integrity and Permeability

**FIGURE 10:**
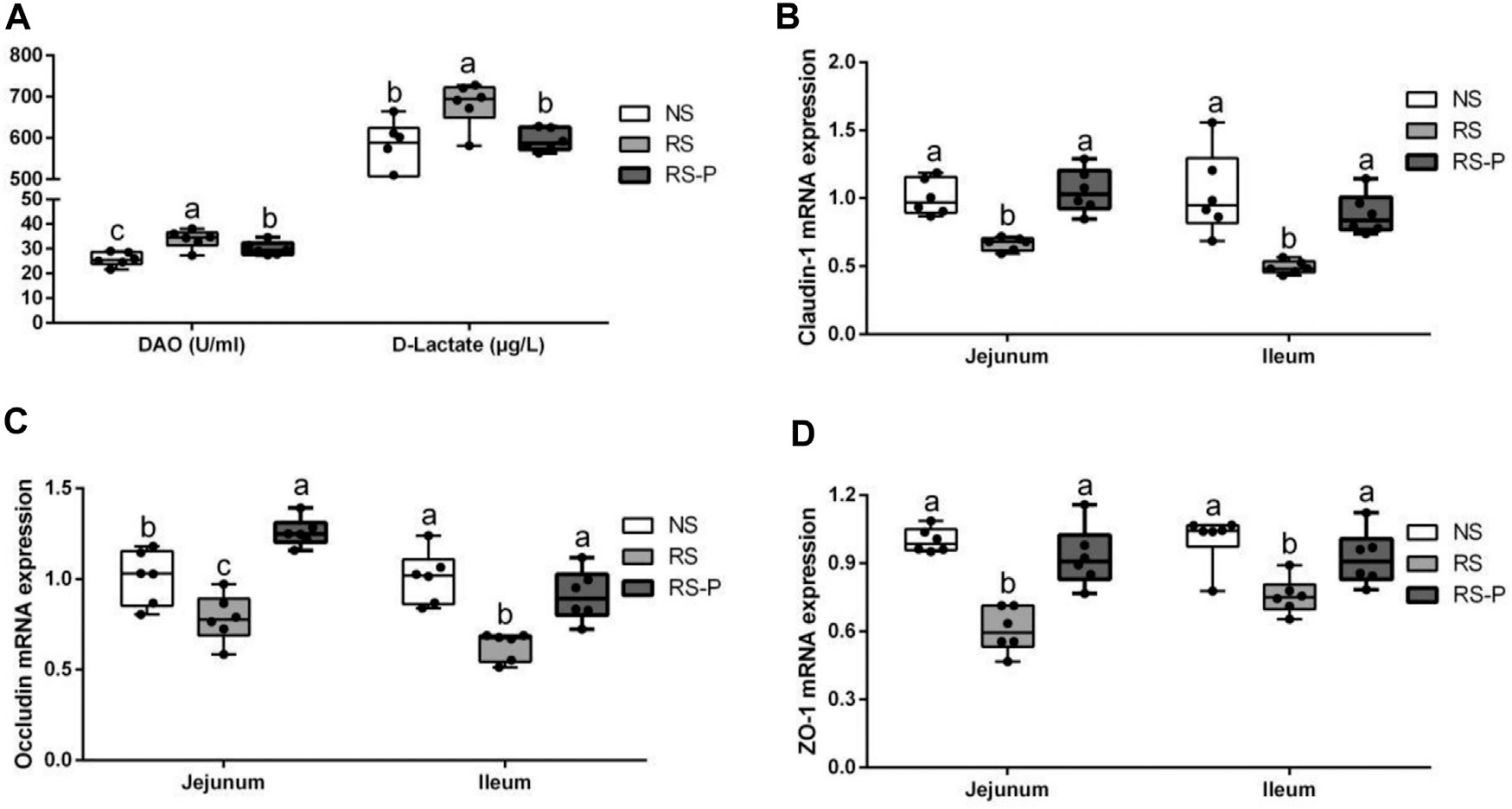
Effect of L. johnsonii BS15 on gut integrity and permeability. Data are presented with the means ± standard deviation (*n* = 6). Bars with different letters are significantly different on the basis of Duncan’s multiple range test (*P* < 0.05). **A**: Levels of DAO and D-Lactate in the serum. **(B)–(D)**: mRNA expression levels of tight junction protein (Claudin-1, Occludin and ZO-1, respectively) in the jejunum and ileum.

**FIGURE 11:**
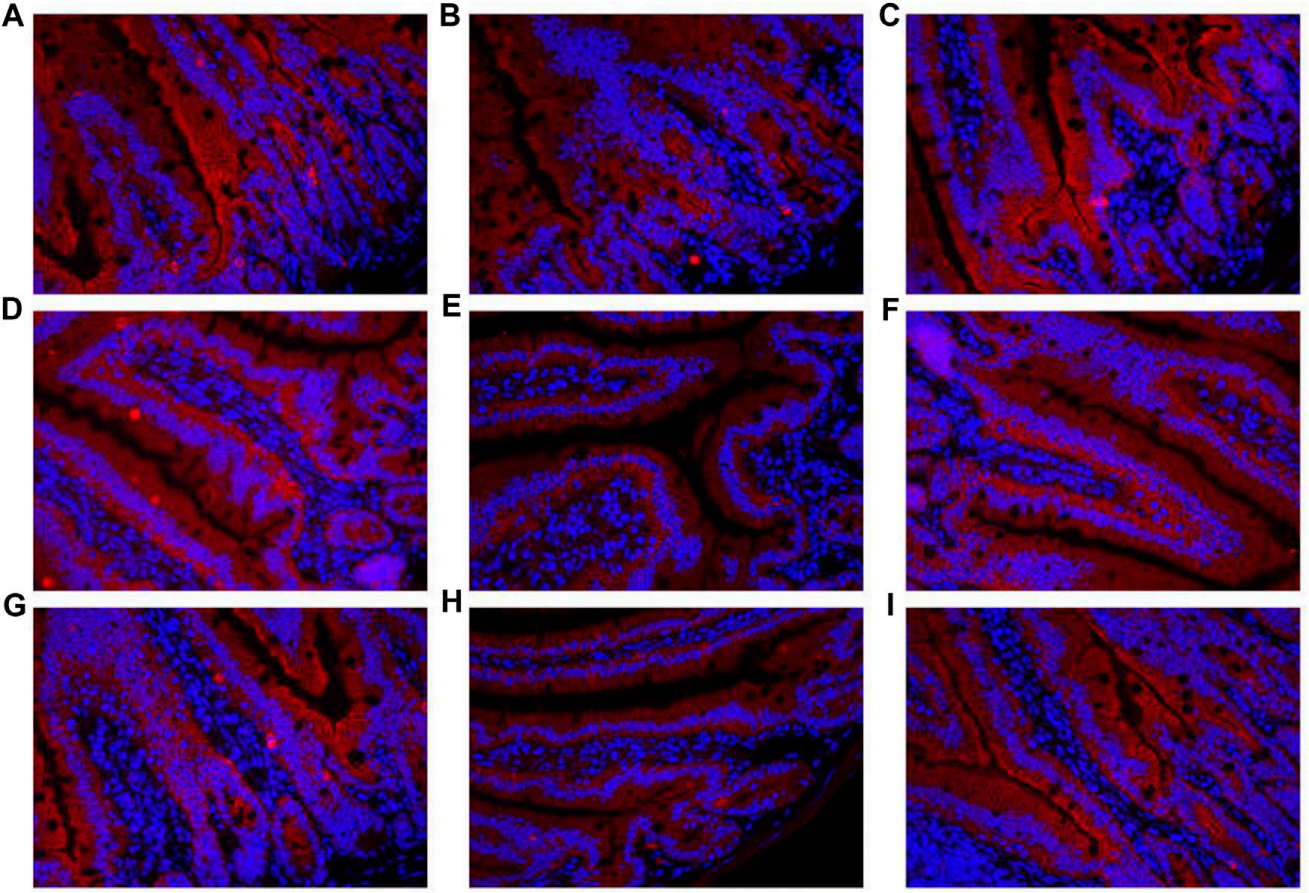
Immunofluorescence of tight junction protein expressions in jejunum of mice. **(A)–(C)** Expressions of Claudin-1 in the jejunum of NS **(A)**, RS **(B)** and RS-P **(C)** groups. **(D)–(F)** Expressions of Occludin in the jejunum of NS **(D)**, RS **(E)** and RS-P **(F)** groups. **(G)–(I)** Expressions of ZO-1 in the jejunum of NS **(G)**, RS **(H)** and RS-P **(I)** groups. The tight junction proteins of. ZO-1-, claudin-1- and occludin are stained red, and the magnification of all the figures are 40×.

**FIGURE 12:**
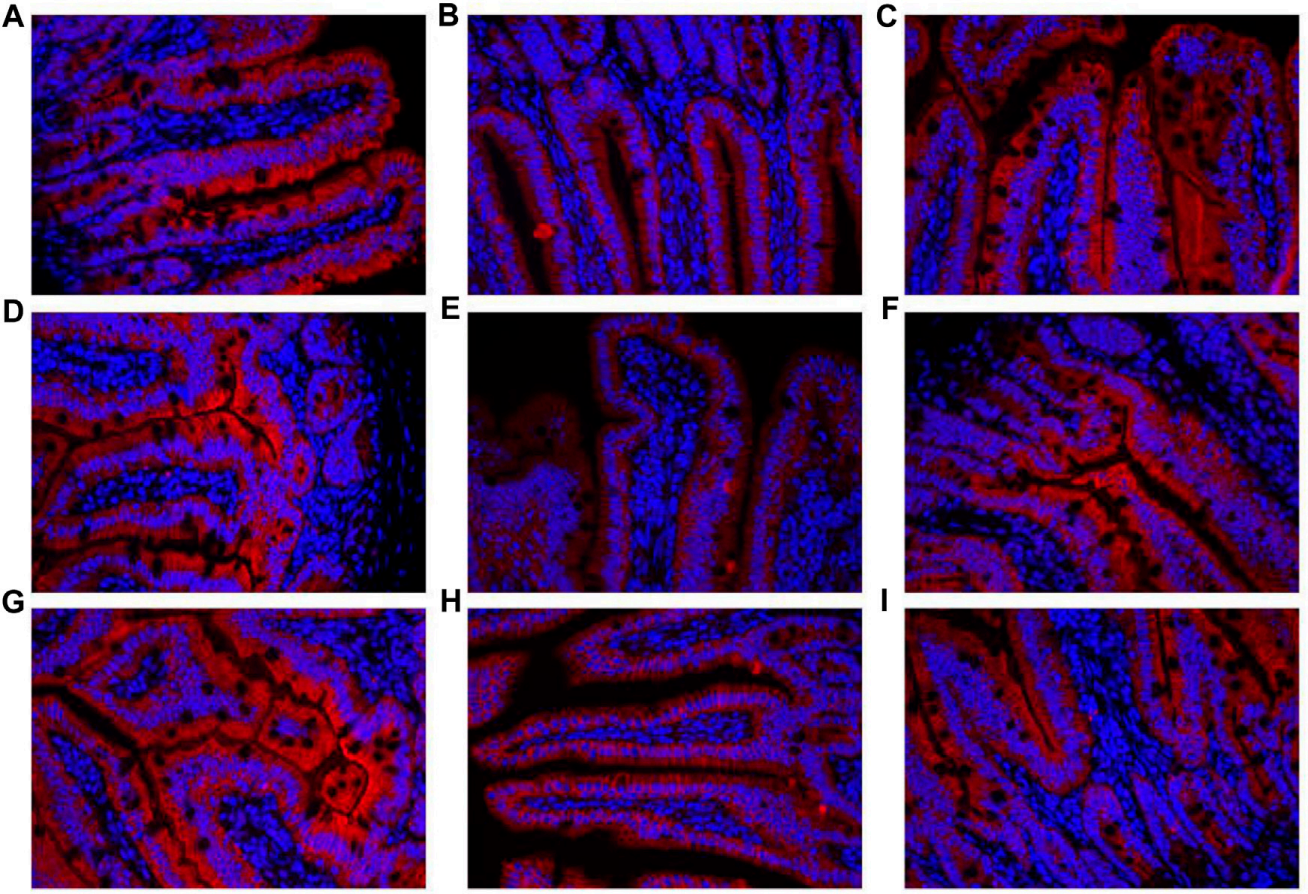
Immunofluorescence of tight junction protein expressions in ileum of mice. **(A)–(C)** Expressions of Claudin-1 in the ileum of NS **(A)**, RS **(B)** and RS-P **(C)** groups. **(D)–(F)** Expressions of Occludin in the ileum of NS **(D)**, RS **(E)** and RS-P **(F)** groups. **(G)–(I)** Expressions of ZO-1 in the ileum of NS **(G)**, RS **(H)** and RS-P **(I)** groups. The tight junction proteins of. ZO-1-, claudin-1- and occludin are stained red, and the magnification of all the figures are 40×.

The contents of DAO and D-lactate in the serum are shown in Figure 10A. The mRNA expression levels of three tight junction proteins in the jejunum and ileum are presented in Figures 10B–D (occludin, claudin-1, and ZO-1). As shown in Figure 10A, the DAO level in the RS-P group was significantly higher (*P* < 0.05) than that in the NS group but lower (*P* < 0.05) than that in the RS group. A significantly higher D-lactate level was observed (*P* < 0.05) in the RS group relative to other two groups. The D-lactate levels showed no differences (*P* > 0.05) between NS and RS-P groups. Except for occludin in the jejunum (Figure 10C) of which the mRNA expression level was higher (*P* < 0.05) than NS group, all mRNA expression levels in jejunum and ileum in the RS-P group were higher (*P* < 0.05) than those in the RS group, but no significant differences (*P* > 0.05) were found between the NS and RS-P groups (Figures 10B–D). The protein expressions of three tight junction proteins were also detected by immunofluorescence, and the results showed the same trend (Figures 11, 12).

### Inflammatory Factors

**FIGURE 13:**
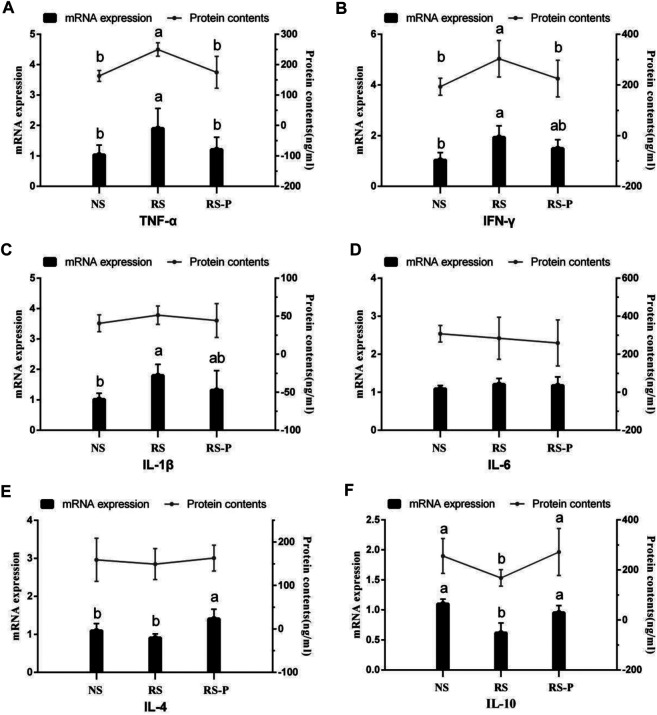
Protein contents and mRNA expression levels of inflammatory factors in the ileum. Data are presented with the means ± standard deviation (*n* = 6). Bars with different letters are significantly different on the basis of Duncan’s multiple range test (*P* < 0.05). **(A)–(F)**: mRNA expression levels and protein contents of TNF-α; IFN-γ; IL-1β; IL-6; IL-4; IL-10, respectively. TNF-α, tumor necrosis factor-alpha; INF-γ, interferon-gamma.

Results of protein contents and mRNA expression levels of inflammatory factors are shown in Figure 13. The inflammatory factors showed significant differences (*P* < 0.05, Figures 13A–F), although a small proportion of the indexes remained unchanged (*P* > 0.05, Figures 13C–E) among the three experimental groups (protein contents of IL-1β, IL-6, and IL-4 and mRNA expression of IL-6). As shown in Figures 13A,B, the mRNA expression levels of TNF-α and IFN-γ and the protein contents of IFN-γ were significantly up-regulated (*P* < 0.05) in the RS group compared with those in the other groups. However, the changes were not controlled by *L. johnsonii* BS15 because no significant differences were detected (*P* > 0.05) between the NS and RS-P groups. In addition, the mRNA expression levels of IFN-γ (Figure 13B) and IL-1β (Figure 13C) in the RS group were significantly higher (*P* < 0.05) than those in the NS group, and these indexes in the RS-P group showed no differences (*P* > 0.05) compared with those in the NS or RS group. Different from the protein content, the mRNA expression level of IL-4 was influenced by *L. johnsonii* BS15 because it was significantly up-regulated (*P* < 0.05) in the RS-P group than that in the NS and RS groups (Figure 13E). Moreover, the protein content and mRNA expression level of IL-10 (Figure 13F) were lower (*P* < 0.05) in the RS group than those in the NS and RS-P groups without significance (*P* > 0.05).

### Gut Microbiota

**FIGURE 14:**
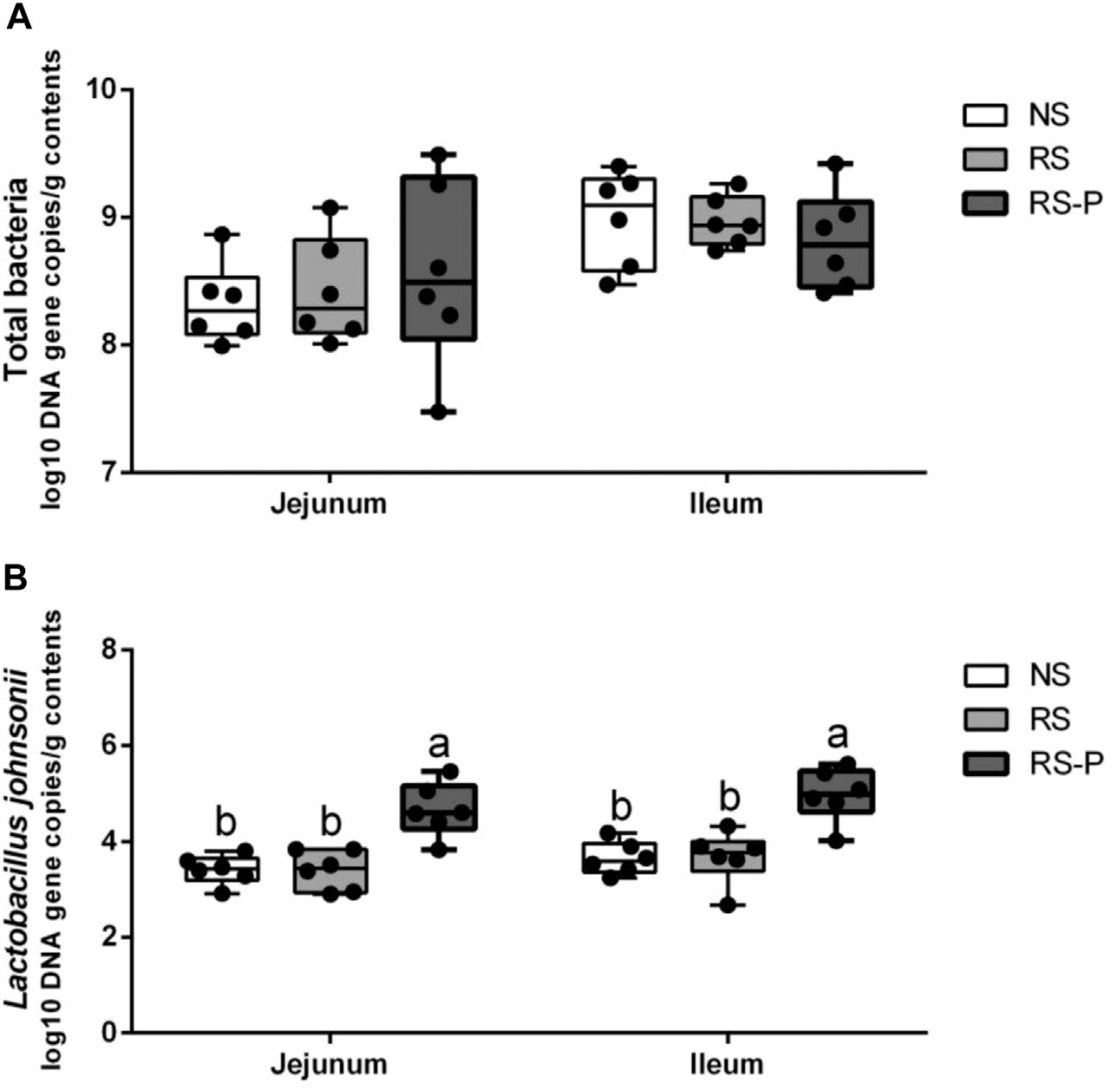
Log10 DNA gene copies of total bacteria **(A)** and Lactobacillus johnsonii **(B)** as quantified by quantitative PCR in the jejunum and ileum. Data are presented with the means ± standard deviation (*n* = 6). Bars with different letters are significantly different on the basis of Duncan’s multiple range test (*P* < 0.05).

Microbial populations in the cecum were quantified via qPCR. The results are presented in Figure 14. The population of total bacteria (Figure 14A) was not significantly different (*P* > 0.05) among all experimental groups in the jejunum and ileum. However, both in the jejunum and ileum, the population of *Lactobacillus johnsonii* (Figure 14B) was significantly higher (*P* < 0.05) in RS-P group than those in NS and RS groups, while no significant difference was observed (*P* > 0.05) between NS group and RS group.

## Discussion

In modern society, psychological stress is common and negatively influences people’s physiological system toward a low utility state (Guo et al., 2017). A quarter of the population in the United States is under high physiological stress (Oken et al., 2015). Stressful events can damage memory performances, such as memory consolidation and memory retrieval. (Mo et al., 2013) reported that increased stress susceptibility in animal models could cause a direct negative effect on memory function. Studies revealed negative influences on the majority of determined indexes, indicating that psychological stress could bring considerable harm to human health. Research achievements in different fields such as psychiatry, gastroenterology, and neuroscience are essential in advancing our understanding of GBA and exploring psychobiotics to regulate the brain by improving gut health, including intestinal inflammation and permeability. Many probiotics have psychobiotic potential of restoring or preventing hippocampal-dependent memory deficits in rodents induced by many different factors such as aging (Jeong et al., 2015) and diabetes (Davari et al., 2013). Although the antinociceptive (Iwakabe et al., 1998) and antidepressant (Ait-Belgnaoui et al., 2009) effects of probiotics on RS-induced mice have been reported, to our best knowledge, limited information is available regarding the preventive effects and underlying mechanism of potential psychobiotics on RS-induced memory dysfunction in rodents.

RS is one of the most widely utilized methods to mimic psychological stress. Rodents are isolated from their group with their movement confined to a restricted area (Bali and Jaggi, 2015; Miyamoto et al., 2017). Sheridan et al., 1991 reported that their immunity against virus is substantially depressed, and IL-2 secretion is reduced in the spleens and mediastinal lymph nodes in RS mice. Similar to many other stressors, RS damages the neuronal morphology and hippocampal function and induces dendritic remodeling in the prefrontal cortex, resulting in the increase in anxiety-like behaviors in humans and animals (Shansky et al., 2009). RS-induced anxiety could disrupt the working memory and therefore is one of the causes of memory dysfunction (Shackman et al., 2006). Stress could be controlled by the activation of the hypothalamic–pituitary–adrenal axis and the subsequent release of stress hormones such as corticosterone (in rodents) that are important for memory ability. The present results revealed increased corticosterone levels in the serum caused by RS and were in agreement with those by Guimaraes and colleagues (1993) and Gregus et al., 2005. In addition, the serum corticosterone level was attenuated by *L. johnsonii* BS15 pretreatment. This phenomenon was associated with the improved performances of the RS-P group during the novel object test, T-maze test, and passive avoidance test. Three different behavioral tests were utilized to evaluate the preventive effects of *L. johnsonii* BS15 on RS-induced hippocampus-related memory dysfunction. The mice in RS-P group were free from damaged memory abilities to some extent, thus suggesting the positive influences of *L. johnsonii* BS15 as a potential psychobiotic strain. The results of total bacteria and *L. johnsonii* population in this study indicated that *L. johnsonii* BS15 is possibly able to colonize in small intestines (jejunum and ileum) of mice, which makes it possible for this probiotic strain to alter the intestinal environment and thus exerts beneficial effects against psychological stress. Although detecting more copies of the genome does not directly demonstrate that the microbe is growing in the gut/metabolically active, as the qPCR approach will amplify genomic matrial from dormant cells, the observed differences between the groups still help to support our hypothesis to some extent.

The hippocampus is a crucial brain area for memory ability and is particularly susceptible to dietary (after less than 1 week) or psychological (30 min of psychological stresses) insult that could cause memory deficits (Guimaraes et al., 1993; Molteni et al., 2002). This work determined the changes of some important memory-related functional proteins, neurotransmitters, antioxidant capacity, and apoptosis-related functional proteins in the hippocampus to reveal the mechanism underlying the promising performances of *L. johnsonii* BS15 in behavioral tests for hippocampus-related memory abilities. Although the exact mechanism of stress-induced memory deficits remains unclear, the decreased neuroplasticity markers in the hippocampus are one of the most important proposed mechanisms (Reichelt et al., 2015).

Brain-derived neurotrophic factor (BDNF) plays an important role in the synaptic plasticity underlying the acquisition and/or consolidation of memory, and the hippocampus-specific deletion of BDNF could cause impaired spatial learning and novel object recognition (Heldt et al., 2007). A substantial decrease in the mRNA expression level of BDNF was found in the RS-induced mice, and this result agrees with the study by Xu et al., 2004. An enteric bacterial infection was reported by Gareau et al., 2011 to impair memory via reduced hippocampal BDNF; *Citrobacter rodentium*-infected mouse showed significant decreases in hippocampal BDNF levels, and reversal in BDNF expression was found in the probiotic-treated group. In addition, gut microbiota damaged by oral antimicrobials in mice reduces the hippocampal mRNA expression of BDNF, and this effect could be reversed by colonizing with normal microbiota (Bercik et al., 2011). A possible link between the significantly up-regulated mRNA levels of BDNF and the improved gut microbiota in RS-P group was suggested because a potentially harmful family of microorganisms, Enterobacteriaceae, was suppressed by *L. johnsonii* BS15. In addition, *Lactobacillus* spp. level also increased, indicating the probable suppression of other non-beneficial bacterial groups (Xin et al., 2014). The change of BDNF level may also be the major cause of the significant up-regulation of CREB. As one of the best-characterized transcription factors in the brain, CREB induced by BDNF is required for various memory forms and plays a role in neuronal resistance to insult in conjunction with BDNF (Gomez-Pinilla et al., 2002). SCF is reported to promote neuronal plasticity (Hutchison et al., 2010). Given that the mRNA expression level of SCF was decreased by RS and effectively prevented by *L. johnsonii* BS15, the results of BDNF, CREB and SCF jointly revealed the close relationship between the preventive effects of *L. johnsonii* BS15 as a psychobiotic and the changes of neuronal plasticity. The results also showed that the RS-induced decreased mRNA expression of c-Fos and NMDAR, two functional proteins closely related to memory formation, were reversibly increased by *L. johnsonii* BS15, thereby suggesting its preventive effects against hippocampal-dependent memory dysfunction. The findings on c-Fos and NMDAR are consistent with the study by Wang et al., 2015 who applied *Lactobacillus fermentum* NS9 to protect the antibiotic-induced physiological and psychological abnormalities in rats.

Liang et al., 2015 pretreated rats with another potential psychobiotic strain, *Lactobacillus helveticus* NS8, and found that the DA and 5-HT contents in the hippocampus were substantially low in the chronic RS-induced group but were enhanced by NS8 pretreatment. Given that 5-HT and NE regulate mood and cognition, the results suggested the therapeutic potential of NS8 through the GBA. Similar results of DA and 5-HT contents in the hippocampus were found in the present study. Other crucial neurotransmitters were also determined. GLU and GABA are important for learning and memory in the hippocampus and serve as excitatory and inhibitory neurotransmitter (Tabassum et al., 2017). Increased Glu content and decreased GABA content commonly indicate damaged hippocampal functions and memory dysfunction. Therefore, *L. johnsonii* BS15 showed beneficial effects by protecting the memory abilities against RS by enhancing GABA content and decreasing Glu content in the RS-P group. Moreover, the preventive effects of *L. johnsonii* BS15 are revealed by the increase in Ach content, which also plays an important role in memory function, especially hippocampus-dependent learning (Haam and Yakel, 2017). Ach is catalyzed by ChAT and removed by the degradative function of AchE. The results indicated that RS could inhibit Ach accumulation by enhancing AchE activity and decreasing ChAT activity, thus damaging the memory function. Based on the results for the RS-P group, the changes of Ach could be prevented by *L. johnsonii* BS15.

Lipid peroxidation is an important process of molecular injury during various oxidative stresses causing hippocampal-dependent memory deficits. The production of reactive oxygen species generated by stress is responsible for lipid peroxidation indicated by increased MDA formation (Niki, 2012). CAT, SOD, and GSH-Px are antioxidant enzymes that protect against oxidative stress by degrading superoxide anions and hydrogen peroxide (Thakare et al., 2017). In this study, RS reduced the activities of T-AOC, GSH-Px, and CAT and increased MDA formation in the hippocampus, suggesting the enhancement of oxidative stress partly associated with the RS-induced memory dysfunction. This finding is consistent with the study by Freitas et al., 2014 and Thakare and colleagues (2017) who obtained similar results in the hippocampus of mice induced by RS for 7 h and 1 h, respectively. Molecular lesions could be induced by oxidative damage inducing and triggering apoptosis. Bcl-2 family proteins are located on the mitochondrial membrane, alter the permeability of mitochondrial membrane, and trigger apoptosis. High vulnerability to apoptotic activation could be indicated by increased Bax and low Bcl-2 (Kasprzak, 1995). The present study found the highest Bax and caspase-3 contents and lowest bcl-2 and Bcl-xL contents in the hippocampus of rodents in the RS group, suggesting that apoptosis mediated by mitochondria is remarkably activated by RS. *L. johnsonii* BS15 also effectively prevented the RS-induced side effects indicated by low Bax and caspase-3 and high Bcl-2 and Bcl-x in the RS-P group. Caspase-dependent apoptotic pathway can be activated by an imbalance between Bcl-2 and Bax, which results in high levels of caspase-3 and -9. Briefly, cytochrome c triggers the association of Apaf-1 to form an apoptosome by leaking out through the holes formed by Bax in the mitochondrial membrane; caspase-9 is activated through the apoptosome, which then triggers caspase-3 activation and consequently causes cell apoptosis (Jarskog et al., 2004). In the present study, the results of apoptosis-related proteins were related to the caspase-dependent apoptotic pathway and revealed that *L. johnsonii* BS15 pretreatment may inhibit oxidative damage in the hippocampus, modulate apoptosis level, and protect mice from RS-induced hippocampal-dependent memory deficits.

One of the most widely accepted mechanisms of how GBA influences cognitive functions is that bacteria in the gut initiate functional signals that are transmitted to the central nervous system through blood circulation. When the gut epithelium tight junctions are impaired, the damaged integrity of intestinal barrier becomes highly permeable, thus allowing the bacteria and/or their metabolites to easily enter the blood circulation (Sgritta et al., 2019). Therefore, the intestinal barrier protective effects of a potential psychobiotic may be the possible mechanisms to prevent mental diseases, including preserving the tight junction protein, inhibiting epithelial apoptosis, decreasing pathogenic bacterial adhesion, and reducing proinflammatory cytokines (Mennigen and Bruewer, 2009). Therefore, gut integrity and permeability were evaluated by determining DAO and D-lactate levels in the serum and the three key tight junction proteins in jejunum and ileum. The levels of DAO and D-lactate, circulating markers for the damage and repair of the intestinal mucosa, reflect the permeability and barrier function in the gut (Liu et al., 2017). The tight conjunction proteins (claudin-1, occluding, and ZO-1) created an intact layer of epithelial cells (Günzel and Yu, 2013). RS remarkably increased the release of DAO and D-lactate in the serum and decreased the mRNA expression levels of all three tight junction proteins, thus reflecting the RS-induced impairment of intestinal barrier. Therefore, *L. johnsonii* BS15 exerted beneficial effects on all determined indexes related to intestinal permeability except for the DAO level possibly through its protection for the intestinal epithelial cell membrane (Zeissig et al., 2007).

Proinflammatory cytokines, such as IFN-γ and TNF-α reduce the epithelial barrier function by influencing the epithelial tight junction and the induction of single cell apoptosis (Uwada et al., 2017). TNF-α and IFN-γ downregulate the mRNA expression of occludin and ZO-1, two tight junction proteins (Mankertz et al., 2000). Zeissig et al., 2004 also found that the damaged integrity of intestinal barrier could be restored to normal in humans by using TNF-α antibody therapy. According to our results, the impairment of intestinal barrier may be associated to the increased proinflammatory cytokines in the ileum. The highest mRNA expression levels of TNF-α, IFN-γ, and IL-1β, three important proinflammatory cytokines, were detected in the RS group, and the same trends of their protein contents (except IL-1β) were also observed. Moreover, IL-10, an anti-inflammatory cytokine, was inhibited by RS. The RS-induced changes were also observed by Gareau et al., 2008 in their study on the relationship between psychological stress and intestinal damage. Although *L. johnsonii* BS15 did not remarkably improve the proinflammatory cytokines, the determined anti-inflammatory cytokines (IL-4 and IL-10) were increased in the RS-P group compared with those in the RS group, indicating that *L. johnsonii* BS15 may induce preventive changes against RS by enhancing the intestinal anti-inflammatory effect and thus maintaining the intestinal integrity. These changes are in agreement with the results reported by Desbonnet et al., 2008 who administered another psychobiotic strain, *Bifidobacterium infantis,* to rats.

In conclusion, the pretreatment of *L. johnsonii* BS15 may prevent RS-induced hippocampus-related memory dysfunction by modulating intestinal inflammation and permeability, which indicated the psychoactive effects of *L. johnsonii* BS15 on positively influencing the GBA.

## Data Availability

The raw data supporting the conclusion of this article will be made available by the authors, without undue reservation.
